# Rectal temperature and heat transfer dynamics in the eye, face, and breast of broiler chickens exposed to moderate heat stress

**DOI:** 10.1016/j.psj.2024.104748

**Published:** 2024-12-30

**Authors:** Moustafa Yehia, Amani Askri, Ahlem Achour, Jean-Michel Allard Prus, Véronique Ouellet, Nabeel Alnahhas

**Affiliations:** a Department of Animal Science, Faculty of Agricultural and Food Sciences, Université Laval, 2425 Rue de l'Agriculture, Quebec City, Quebec G1V 0A6, Canada; b Scott Hatchery, 1798 Rue du Président Kennedy, Scott, Quebec G0S 3G0, Canada; c Swine and Poultry Infectious Diseases Research Center, Faculty of Veterinary Medicine, Université de Montréal, 3200 Rue Sicotte, St-Hyacinthe, Quebec J2S 2M2, Canada

**Keywords:** Broiler chickens, Hyperthermia, Infrared thermography, Rectal temperature, Skin temperature

## Abstract

This study aimed to characterize body temperature in finishing broiler chickens and to explore heat transfer dynamics under thermoneutral (**TN**) and heat stress (**HS**) conditions. To achieve this, 900 Ross 308 chicks were divided into TN and HS groups, with the HS group subjected to cyclical heat stress (30°C, 45 % RH) from day 28 to day 33 post-hatch. Rectal temperature (T_r_) and skin temperature (T_s_) at the face (T_sf_), eye (T_se_), and breast (T_sb_) were measured. T_r_ ranged from 39.1 to 40.6°C under TN and from 40.4 to 43.2°C under HS (*P*_condition_ < 0.001). Core-to-skin temperature gradients were lower under HS (*P* < 0.0001), indicating reduced heat transfer to the skin. All T_s_ parameters were higher under HS (*P* < 0.001), and skin-to-air temperature gradients were also lower (*P* < 0.0001), reflecting lower heat dissipation. T_s_ varied significantly across anatomical regions (*P* < 0.001), and core-to-skin and skin-to-air gradients differed between regions under both conditions (*P* < 0.001). Strong correlations were observed between T_r_ and T_s_ (*r* = 0.88, 0.89, and 0.92 for T_sb_, T_se_, and T_sf_, respectively), suggesting T_s_ as a strong predictor of T_r_. In conclusion, rectal temperatures in finishing broilers are more variable under HS than under TN. Under HS, some birds continue to exhibit T_r_ in the physiological range. Under TN, heat is transferred from the core to the eyes at a significantly higher rate than to the face and breast skin. However, the breast skin dissipates heat into the environment at a greater rate than the face and the eyes. These patterns of heat transfer between the core and the skin, and between the skin and the environment are conserved under HS. However, heat transfer rates are significantly reduced leading to increased heat load of the birds. These findings provide further insights into thermoregulation in broiler chickens.

## Introduction

To continue to meet the increasing global demand for broiler meat, the poultry industry leverages genetic selection to create and develop broiler strains with faster growth rates, higher feed efficiencies, and greater breast meat yield (Zuidhof et al., 2014). However, accelerated growth is associated with increases in the metabolic rate and heat production (Nascimento et al., 2017). These selection-induced changes in heat production, alongside factors such as the inherent absence of sweat glands and the insulating capacity of feathers, exacerbate broiler chickens’ susceptibility to HS (Malila et al., 2021; Tabler et al., 2020). Consequently, HS has become one of the important challenges that the poultry industry currently faces. This is especially true in the context of continuously increasing surface temperatures due to global warming. It is therefore expected that the economic consequences of HS for the poultry industry will intensify in the coming years (Kumar et al., 2021). The consequences of HS on broiler production are wide-ranging, including deteriorations in broiler performance (Andretta et al., 2021), health (Alhenaky et al., 2017), welfare (Cartoni Mancinelli et al., 2023), and product quality (Malila et al., 2021), which explains the continued gain in research interest over the past two decades (Uyanga et al., 2023).

Conducting research on avian thermoregulation requires repeated measurements of core body temperature, often achieved using a rectal thermometer (Han et al., 2019). This method is invasive and time-consuming, additionally stressing the birds. Alternative methods include telemetry-based temperature loggers (that birds swallow), although they are limited in terms of battery life, large-scale monitoring capacity, and require bird slaughter for recovery (Brown–Brandl et al., 2003; Purswell et al., 2012). Infrared thermography is a non-invasive method for measuring skin temperature, and it correlates strongly with core body temperature, allowing for accurate predictions (Giloh et al., 2012; McCafferty, 2013).

One frequent observation in the literature is that most studies report only average body or average skin temperatures under different environmental conditions. Few studies report the full range of temperature variability, which limits our understanding of broiler chickens' ability to cope with high ambient temperatures. Understanding the full range of body temperature under thermoneutral conditions is crucial for making accurate statements about thermotolerance under heat stress. Additionally, most studies using thermography focus solely on facial temperatures, which limits our understanding of skin-to-environment heat transfer since different anatomical regions would be expected to contribute differentially to heat dissipation.

In a previous study, we used infrared thermography to measure the facial temperature of finishing broilers kept under TN and severe cyclical HS conditions (Yehia et al., 2024). We observed that facial temperatures under HS conditions were more variable than under TN conditions in both males (average ± std: 38.2 ± 0.78 vs 42.7 ± 1.2°C under TN and HS, respectively) and females (average ± std: 38.0 ± 0.81 vs 42.6 ± 1.2°C under TN and HS, respectively), with the standard deviation of facial temperatures being 1.5 times higher under HS than under TN conditions. In the current study, we built on findings from our previous study and hypothesized that bird core temperature would be more variable under HS conditions than under TN conditions. We further hypothesized that under both HS and TN different anatomical regions of the skin are prioritized differently for heat dissipation. We evaluated this hypothesis through: (1) characterizing and comparing the range of variation in broiler core body temperature under TN and moderate cyclical HS conditions during the finisher phase (d 28 – d 34), (2) investigating the relationship and heat transfer dynamics between core body temperature and skin temperature as measured at different anatomical regions under these conditions, and (3) elucidating the impact of HS on this relationship and on heat transfer dynamics.

## Materials and methods

All experimental and animal care procedures were reviewed and approved by the Institutional Animal Care and Use Committee of Université Laval according to the guidelines of the Canadian Council on Animal Care (Project #2022-1016).

This experiment is part of a larger ongoing project investigating HS in finishing broiler chickens. In May 2024, the first study on the effects of severe cyclical HS on broiler chicken performance and oxidative status was published (Yehia et al., 2024). In the study presented here, a moderate cyclical HS program was used to simulate moderate summer conditions without severe heat waves.

### Birds and housing

A total of 900 one-day-old Ross 308 chicks (*n* = 450 per sex) were acquired from a commercial hatchery (Scott Hatchery, Scott, Quebec, Canada) and placed in an experimental poultry house at the Deschambault Research Center in Animal Science (Deschambault, Quebec, Canada) between November and December 2023. As described by Yehia et al. (2024), the house was divided into two separate sections or rooms: a TN (control) and HS room. Both sections were equipped with floor pens (*n* = 10 pens per room, with 5 pens per sex, and a total of 45 birds per pen for a final rearing density of 31 kg/m^2^). The floor pens (3.6 m^2^/pen) were bedded with sawdust and equipped with bell drinkers and manual feeders.

### Experimental design

**Fig. 1:**
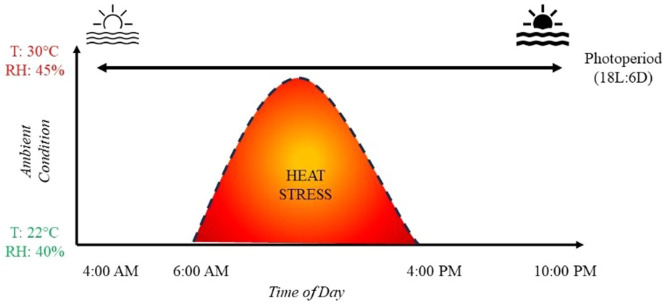
An illustration of the heat stress program applied in the current study in relationship to the photoperiod. The photoperiod started at 4:00 AM and ended at 10:00 PM while the heat stress period started at 6:00 AM and ended at 4:00 PM.

From d 1 to d 27, birds in all pens in both sections of the poultry house were kept under the same environmental conditions corresponding to their requirements. The ambient temperature was maintained at 33°C during the first week post-hatch, gradually reduced to 22°C by the end of the third week, and then maintained at 22°C up to d 27 of the experiment. On d 28, birds placed in the control room continued to be kept under TN (22°C, 45 % relative humidity) conditions. As for birds placed in the stress room, they were exposed to a cyclical HS program up to the end of the experiment on d 34. The HS program consisted of increasing the ambient temperature to 30°C while the relative humidity was maintained between 40 and 45 % from 6:00 AM to 4:00 PM (Fig. 1). At the end of the daily HS program, the environmental conditions were returned to TN. During the stress period, ambient temperature (T_a_) and relative humidity (RH) were recorded hourly using temperature loggers (RuuviTag Bluetooth Sensor, Finland) installed at the level of the birds in both sections of the poultry house (*n* = 3 per section). Regarding the lighting program, the photoperiod was set to 23 h per day for the first 4 days to allow the chicks to discover their environment and to identify the drinkers and feeders. It was then gradually reduced to 18 h of light and 6 h of darkness (18L:6D) per day. This lighting program was then maintained until the end of the experiment. The photoperiod started at 4:00 AM and ended at 10:00 PM (Fig. 1). Throughout the entire experimental period, water and feed were available *ad libitum*. Birds received a standard commercial broiler starter (d 1 to d 11), grower (d 12 to d 21), and finisher (d 22 to d 34) diets. Diet formulation and nutrient contents were the same as described previously (Yehia et al., 2024).

### Performance traits

Performance traits of importance for understanding thermodynamic data including bird body weight (BW), average daily feed intake (ADFI), and feed conversion ratio (FCR) were recorded before (d 28) and after (d 34) of the application of the HS program.

### Temperature measurement

In each pen, three birds were randomly selected and marked on the back with a non-toxic colouring agent. Temperature measurements were repeatedly performed on these same three birds throughout the duration of the experiment (*n* = 30 birds per condition).

### Rectal temperature (T_r_)

Body temperature (T_b_) was recorded by measuring bird rectal temperature (T_r_) using a digital thermometer (AG-102 Animal Thermometer, AG-Medix, Wisconsin, USA) that was inserted 3 cm into the rectum through the cloaca and held in the rectum until the temperature reading was stabilized. This thermometer was previously calibrated against a standard mercury thermometer to be accurate to ± 0.1°C. To minimize handling stress during temperature recording, the measurements were performed by two trained operators. One operator held and oriented the bird with both hands while the other operator performed the measurements. T_r_ of the birds was recorded twice a day (once between 9:00 and 10:45 AM and once between 2:00 and 3:45 PM) from d 28 to d 33.

### Skin temperature (T_s_)

**Fig. 2:**
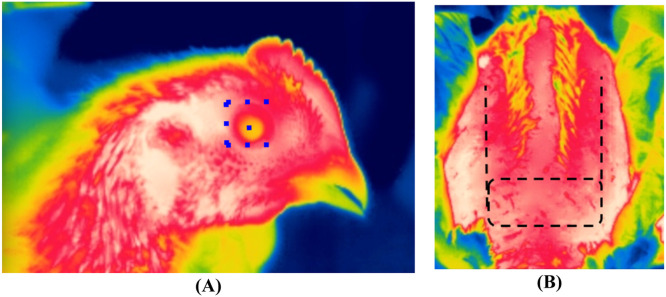
Infrared thermal images illustrating the anatomical regions where facial, eye (A) and breast skin (B) temperatures were recorded. The facial temperature was recorded along the three dotted lines around the eye and an average over the three lines was then taken as the final facial temperature. The eye temperature was measured exactly in the center of the eye (blue square). The breast temperature was measured in the black dashed box and along the two black dashed lines and the average of these readings was taken as the final temperature of the breast skin.

T_s_ was measured only on d 33 of the experiment on the same three marked birds per pen using an infrared thermal camera (model T440bx, FLIR Systems). This camera is equipped with a high-resolution (320 × 240 pixels) lens capable of measuring temperatures within the range of −20 to 650°C with 2 % accuracy, which is typical of thermal cameras used in studies on livestock (McManus et al., 2022). The emissivity parameter of the thermal camera was set at 0.95, which is within the range reported in the literature on the use of thermography in livestock (McManus et al., 2022). T_s_ was recorded at three anatomical regions: the face excluding the eye (T_sf_), the eye (T_se_), and the breast (T_sb_). The bird was first placed on a clean wheeled table and allowed to settle prior to temperature measurement. To measure T_sf_ and T_se_, a thermal image of the bird's face was taken (side view) with a lens distance of 10 cm (Fig. 2A). For T_sb_ measurements, one operator held the bird in the supine position with both hands, breast facing upward (bottom view), while the second operator took the thermal image of the anatomical region between the cranial section of the pectoral muscles and the apex of the sternum at a distance between the camera and the birds allowing for the capture of this anatomical region (Fig. 2B). This region represents the largest surface area where the skin encounters the environment (i.e., air and litter), helping birds to dissipate body heat. Thermal images were then processed using the FLIR Thermal Studio software (FLIR Systems) to determine the temperature reading in the featherless (i.e., the skin) facial and breast regions, as well as the center of the eye (Fig. 2). For T_sf_, the average temperature around the eye (blue square lines) was used, while T_se_ was measured directly at the center (single blue square) of the eye (Fig. 2A). T_sb_ was calculated based on the average temperature of the highlighted (black dashed lines) section of the breast (Fig. 2B).

### Temperature gradients (∇T)

Temperature gradients (∇T) between T_r_ and T_s_ (T_sf_, T_se_, and T_sb_) were calculated according to previously published equations (Lin et al., 2005b). Briefly, ∇T between T_r_ and T_s_ was calculated as follows:

$$\nabla T_{r-s}=\;T_{r}-\;T_{s}$$

Similarly, ∇T between the skin temperature (T_sf_, T_se_, and T_sb_) and the ambient temperature (T_a_) was calculated as follows:

$$\nabla T_{s-a}=\;T_{s}-\;T_{a}$$

### Statistical analysis

Descriptive statistics of T_r_ and T_s_ were computed using R (R Core Team, 2020). The effects of condition (TN, HS), sex, and their interaction on the different measurements of bird temperatures and their gradients were analysed using a linear mixed effects model as implemented in the *lmerTest* package (Kuznetsova et al., 2017) of R version 4.0.2 (R Core Team, 2020). The model included the thermal condition, sex, age (only for T_r_), time of measurement during day (AM/PM, only for T_r_), and their interactions as fixed effects, while the effects of the room (i.e., section of the poultry house) and the pen-intra-room were fitted as random effects to account for potential random variability in environmental conditions. To investigate the effect of the anatomical region of measurement on temperature readings obtained through infrared thermography, a similar model that included the anatomical region (face, eye, breast) was also fitted to the data. For performance traits, they were analysed using the same statistical model that included thermal conditions, sex and their interaction as fixed effects. Results were reported as least squares means and their standard errors. Differences between group means were tested for significance using the Tukey method as implemented in the *emmeans* package of R. Finally, to understand the change in the relationship between T_r_ and T_s_ under different environmental conditions, pairwise Pearson correlation coefficients between these parameters (measured on the same birds on d 33) were estimated and tested for significance using the *cor.test()* function of R (R Core Team, 2020). Correlation coefficients between temperature parameters obtained under different environmental conditions (*n* = 30 birds/parameter/condition) were tested for significant differences using the Fisher's z-test for differences of correlations in two independent samples as implemented in the *diffcor* package of R using the function *diffcor.two()*. The results were reported as z values and their corresponding *P*-values. For all statistical tests, the significance threshold was set at *P* < 0.05.

## Results

**Fig. 3:**
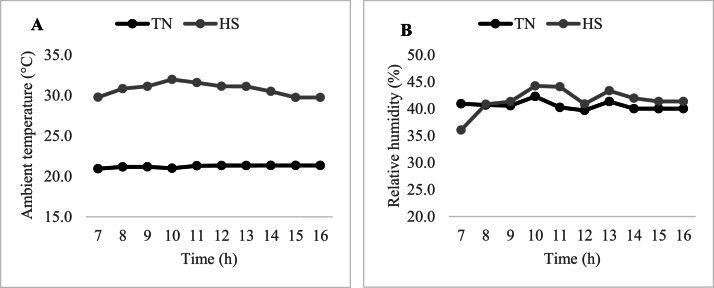
Average hourly ambient temperature (A) and relative humidity (B) between d 28 and d 33 in the heat stress (HS) and thermoneutral (TN) sections of the poultry house.

Average hourly T_a_ and RH in the HS and TN sections of the house during the application of HS are presented in Fig. 3A and 3B, respectively.

### Performance traits

The effect of sex on BW and FCR was statistically significant on d 28, prior to the application of the HS program (Table 1). Males had higher BW (+207 g, P < 0.001) and lower FCR (−0.22 points, *P* = 0.01) compared to females, while ADFI remained similar between sexes.

**Table 1:** Effect of sex on performance traits before the application of the heat stress program (d 28).

Trait^1^	Males	Females	SEM	Sex
BW, g	1921.0	1714.0	12.40	< 0.001
ADFI, g/d/bird	142.0	146.0	6.67	0.70
FCR	1.18	1.40	0.05	0.01

1 Values are the least squares means of 10 pens per group. BW: body weight, ADFI: average daily feed intake, FCR: feed conversion ratio.

At the end of the HS phase on d 33, the sex-by-condition interaction was not statistically significant (Table 2). As can be seen in this table, males continued to have significantly higher BW than females (+293 g, *P* < 0.001). Males also had a significantly higher ADFI than females (+32 g/d, *P* < 0.001), although FCR did not differ significantly between the two sexes.

**Table 2:** Effect of condition and sex on performance traits at the end of the stress phase (d 34).

Traits^1^	Condition^2^				Sex				*P*-Value		
	TN	HS	SEM		Males	Females	SEM		Condition	Sex	I^3^
BW, g	2416.0	2235.0	22.00		2472.0	2179.0	22.00		< 0.001	< 0.001	0.24
ADFI, g/d/bird	169.0	150.0	4.080		175.0	143.0	4.08		< 0.01	< 0.001	0.44
FCR	2.090	2.690	0.160		2.45	2.33	0.160		0.01	0.58	0.47

1 Values are the least squares means of 10 pens per group. BW: body weight, ADFI: average daily feed intake, FCR: feed conversion ratio.

2 TN: thermoneutral conditions (22°C and 45 % relative humidity), HS: heat stress conditions (30°C, 45 % relative humidity for 10 h/day).

3 P-Value of the condition-by-sex interaction.

Regarding the effect of HS, the application of the moderate HS program led to a significant decrease in BW (−7.5 % or 181 g, *P* < 0.001), in ADFI (−11.2 % or 19 g/d, *P* < 0.01), and to a significant increase in FCR (+28.7 % or 0.6 points, *P* = 0.01) compared to TN conditions (Table 2).

### Rectal temperature (T_r_)

**Fig. 4:**
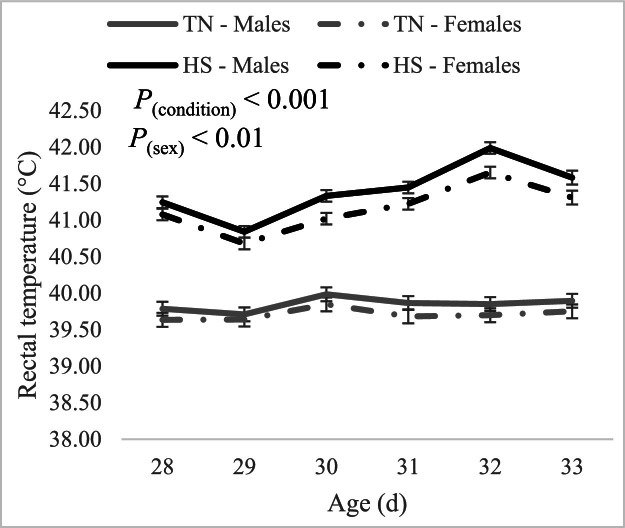
Effect of experimental conditions and sex on rectal temperature of finishing broiler chickens during the heat stress period. TN: thermoneutral (22°C, 45 % relative humidity), HS: heat stress (30°C, 40 to 45 % relative humidity).

**Fig. 5:**
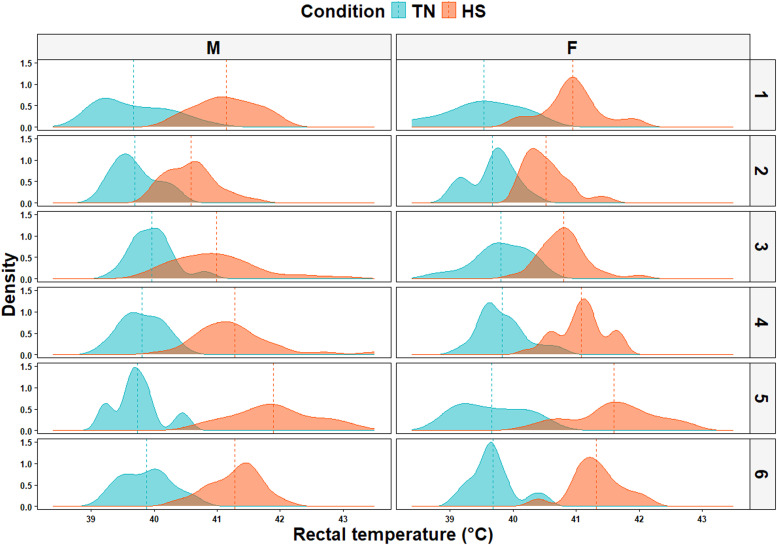
Distribution of rectal temperatures of birds kept under thermoneutral (TN) conditions (22°C, 45 % relative humidity) and heat stress (HS) conditions (30°C, 40 to 45 % relative humidity) over the six days experimental period (*n* = 30 birds/condition/day).

Descriptive statistics of T_r_ are presented in Table 3. Under TN conditions, average T_r_ ranged between 39.1 and 40.6°C (ΔT_r_ = 1.5°C), while it ranged between 40.4 and 43.2°C (ΔT_r_ = 2.8°C) under HS conditions. In the current study, neither the effect of the sex-by-condition interaction (*P* = 0.45) nor that of the sex-by-day of stress interaction (*P* = 0.75) on T_r_ was significant. However, the statistical analysis revealed that the main effect of sex and condition on T_r_ were significant (*P* < 0.01 and *P* < 0.001 for sex and condition, respectively). As can be seen in Fig. 4, birds that were exposed to HS had significantly higher T_r_ compared to birds that were kept under TN conditions (41.28 ± 0.04°C vs 39.78 ± 0.04°C). Additionally, males had a slightly but significantly higher T_r_ than females (40.63 ± 0.04°C vs 40.43 ± 0.04°C). For both males and females, T_r_ of some birds kept under HS overlapped with those of birds kept under TN (Fig. 5).

**Table 3:** Descriptive statistics of rectal temperature (°C) of broilers kept under thermoneutral and heat stress conditions during the finisher phase.

Condition^1^	Sex	Mean	SD	Min	Max	CV (%)
TN	Males	39.90	0.32	39.30	40.60	0.81
	Females	39.80	0.37	39.10	40.50	0.93
HS	Males	41.60	0.59	40.40	43.20	1.41
	Females	41.30	0.41	40.40	42.10	0.99

1 TN: thermoneutral (22°C, 45 % relative humidity), HS: heat stress (30°C and 40 – 45 % relative humidity, 10 h/day from d 28 to d 33).

### Skin temperature (T_s_)

Descriptive statistics of temperature data extracted from thermal images of the face, eye, and breast region are reported in Table 4. Skin temperature readings obtained by infrared thermography (T_s_) exhibited a wider range of values compared to T_r_. Under TN conditions, T_se_ exhibited the widest range of values (ΔT_s_ = 4.7°C, 3.04°C, and 3.25°C for T_se_, T_sf_, and T_sb_, respectively), while under HS conditions, T_sb_ had the widest range of values (ΔT_s_ = 2.7°C, 2.66°C, and 5.34°C for T_se_, T_sf_, and T_sb_, respectively).

**Table 4:** Descriptive statistics of skin^1^ temperature (°C) obtained by infrared thermography of finishing broilers kept under thermoneutral and heat stress conditions.

Condition^2^	Sex	Mean	SD	Min	Max	CV (%)
		**Facial temperature**				

TN	Males	36.20	0.78	35.10	37.90	2.14
	Females	36.50	0.77	34.80	37.40	2.13
HS	Males	40.40	0.75	38.50	41.10	1.86
	Females	39.90	0.76	38.70	41.10	1.89

		**Eye temperature**				

TN	Males	33.20	0.92	31.40	35.20	2.76
	Females	33.20	1.31	31.50	36.10	3.93
HS	Males	37.00	0.52	36.10	37.80	1.40
	Females	36.80	0.86	35.10	37.80	2.34

		**Breast temperature**				

TN	Males	37.80	0.87	36.30	38.90	2.29
	Females	37.80	1.07	35.90	39.20	2.82
HS	Males	41.90	1.42	38.20	43.60	3.39
	Females	42.10	0.88	40.60	43.40	2.09

1 The facial, eye and breast temperatures are defined in Fig. 2 and were measured on d 33.

2 TN: thermoneutral (22°C, 45 % relative humidity), HS: heat stress (30°C and 40 – 45 % relative humidity, 10 h/day from d 28 to d 33).

**Fig. 6:**
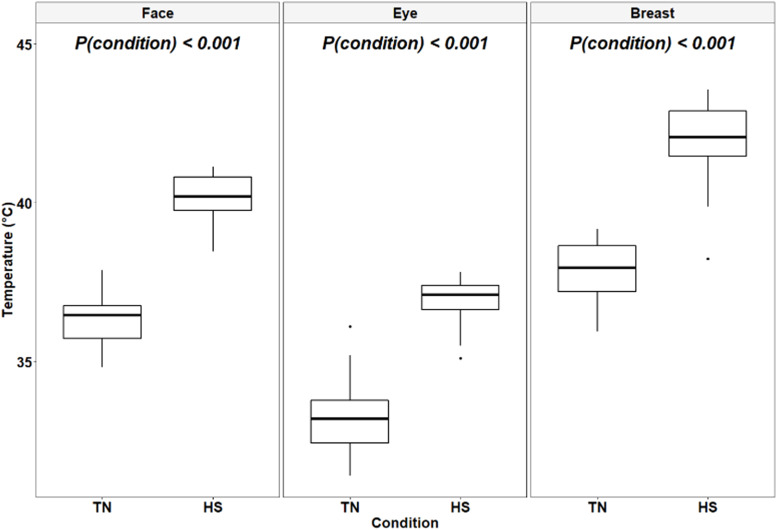
Effect of experimental conditions on the facial, eye and breast temperatures obtained using infrared thermography of finishing broiler chickens. TN: thermoneutral (22°C, 45 % relative humidity), HS: heat stress (30°C, 40 to 45 % relative humidity).

Regarding T_s_, the effect of the sex-by-condition interaction on T_sf_, T_se_, and T_sb_ was not significant (*P* = 0.85, *P* = 0.66, and *P* = 0.94, respectively). Moreover, the main effect of sex was not significant either (*P* = 0.21, *P* = 0.82, and *P* = 0.82 for T_sf_, T_se_, and T_sb_, respectively). However, the statistical analysis revealed a highly significant effect of experimental conditions on T_s_ parameters (*P* < 0.001 for T_sf_, T_se_, and T_sb_), with birds that were exposed to HS having higher T_s_ values (Fig. 6).

**Fig. 7:**
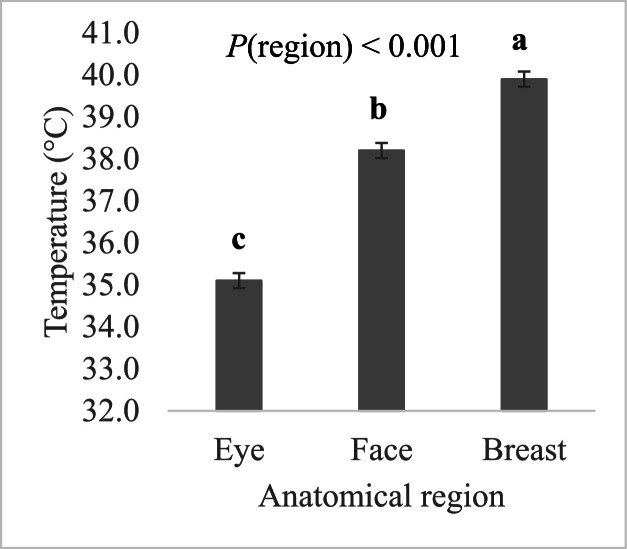
Effect of the anatomical region on skin temperatures obtained by infrared thermography in finishing broiler chickens.

**Fig. 8:**
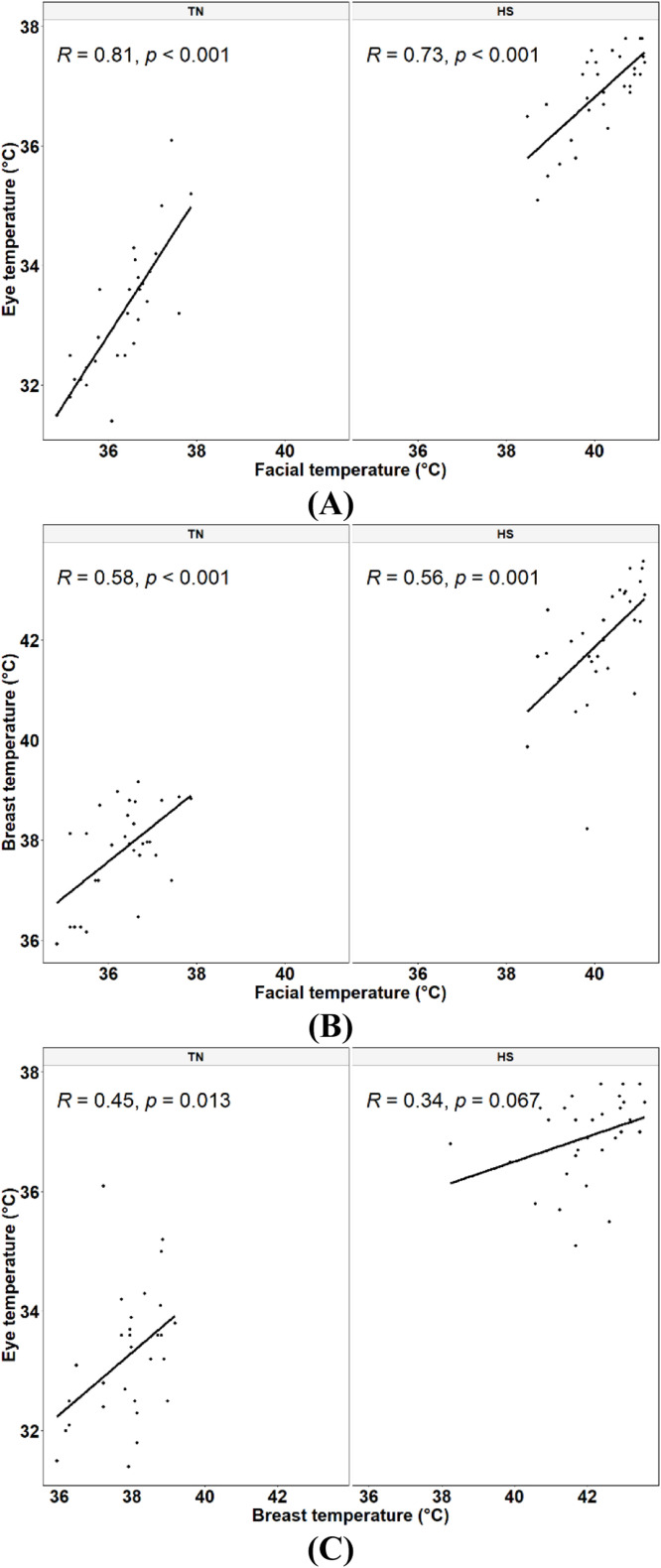
Correlation coefficients between the facial temperature and the eye temperature (A), between the facial temperature and the breast skin temperature (B) and between the breast skin and the eye temperatures (C) under thermoneutral (TN, 22°C, 45 % relative humidity) and heat stress (HS, 30°C, 40 to 45 % relative humidity) conditions (*n* = 30 birds).

In this study, we also compared thermal temperature readings collected from these three distinct anatomical regions. The statistical model that included the anatomical region as a fixed effect revealed that temperature readings differed significantly between these regions (*P* < 0.001), with T_se_ < T_sf_ < T_sb_ (Fig. 7). On the other hand, the effect of condition-by-region interaction on T_s_ was not statistically significant (*P* = 0.14). Despite the variation in T_s_ between different anatomical regions, all three parameters were significantly correlated. Fig. 8 presents the correlations between T_sf_, T_se_, and T_sb_ under TN and HS conditions. As expected, the T_sf_ and T_se_ were strongly and significantly correlated under both conditions (Fig. 8A), with the correlation between these two parameters under TN conditions being slightly but not significantly higher than that under HS conditions (*z* = 0.73, *P* = 0.23). As for T_sb_, it was significantly correlated with both T_sf_ (Fig. 8B) and T_se_ (Fig. 8C) under TN conditions. However, it was only significantly correlated with T_sf_ under HS conditions. The correlations between T_sf_ and T_sb_ were almost identical under both environmental conditions as evidenced by the Fisher's z-test (*z* = 0.11, *P* = 0.45). As for the correlation between T_se_ and T_sb_, it was moderate under TN and low under HS conditions, but the correlation coefficients between these two parameters under different environmental conditions were not statistically different (*z* = 0.48, *P* = 0.32).

**Fig. 9:**
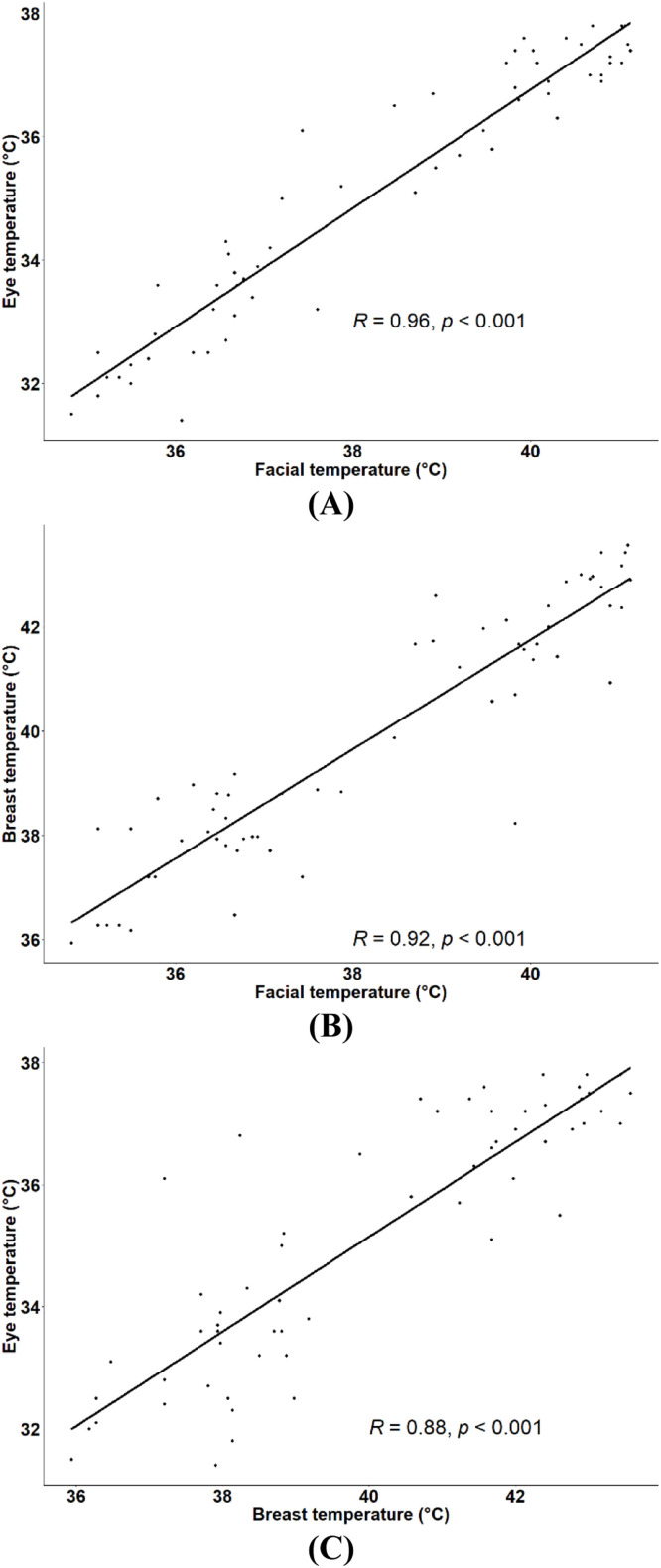
Correlation coefficients between the temperature of the face and the eye (A), between the temperature of the face and the breast skin (B) and between the breast skin and the eye temperatures (C). Data obtained under thermoneutral (22°C, 45 % relative humidity) and heat stress (30°C, 40 to 45 % relative humidity) conditions were combined together (*n* = 60 birds).

Finally, when temperature data obtained under TN and HS conditions were combined together, very strong and highly significant correlations ranging from 0.88 to 0.96 were found (Fig. 9).

### The relationship between T_r_ and T_s_

**Fig. 10:**
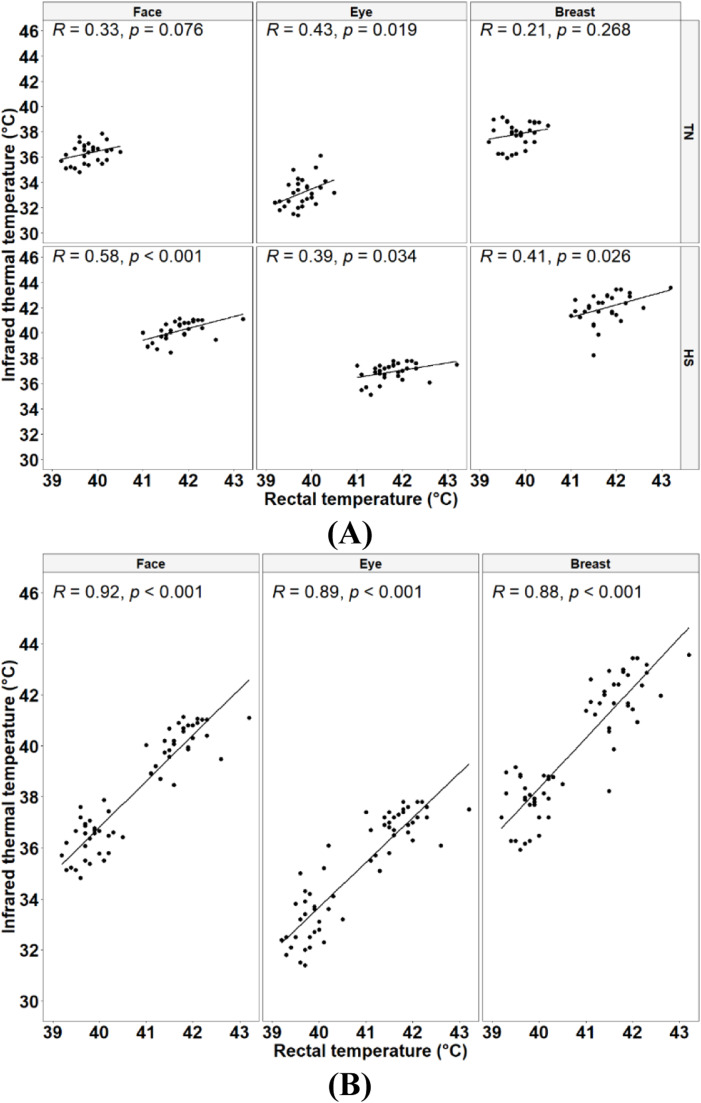
Correlation coefficients between rectal and skin temperatures under thermoneutral (TN, 22°C, 45 % relative humidity) and heat stress (HS, 30°C, 40 to 45 % relative humidity) conditions (A, *n* = 30 birds per condition), and between rectal and skin temperature parameters aggregated over thermoneutral and heat stress conditions (B, *n* = 60 birds).

As illustrated in Fig. 10, the strength of the correlation between T_r_ and T_s_ varied depending on the environmental condition. Under TN conditions, the correlations were low to moderate, ranging from 0.21 to 0.43 (Fig. 10A, upper panel). Only the correlation between T_r_ and T_se_ was statistically significant, while the correlations between T_r_ and T_sf_ and between T_r_ and T_sb_ were not significant. Nevertheless, under HS conditions, correlations were moderate to high, ranging from 0.39 to 0.58, all being statistically significant (Fig. 10A, lower panel). However, Fisher's z-test did not reveal any significant difference between the correlation coefficients obtained under TN conditions compared to their HS counterparts (*P* = 0.12, *P* = 0.43 and *P* = 0.21 for T_sf_, T_se_, and T_sb_, respectively). Interestingly, when temperatures obtained under both conditions were combined (Fig. 10B), very strong correlations between T_r_ and T_s_ (T_sf_, T_se_, and T_sb_) readings were observed, ranging from 0.88 to 0.92.

### Temperature gradients (∇T)

Under HS conditions, both core-to-skin and skin-to-air gradients significantly decreased as compared to TN conditions (Table 5). The statistical analysis showed that when compared intra-condition, all ∇T differed significantly amongst themselves (*P* < 0.001). As can be seen in Table 5, core-to-skin gradients (∇T_r-s_) exhibited the trend of ∇T_r-sb_ < ∇T_r-sf_ < ∇T_r-se_, while skin-to-air gradients (∇T_s-a_) followed the trend of ∇T_se-_*_a_* < ∇T_sf-_*_a_* < ∇T_sb-a_ under both conditions. Interestingly, these exact trends were observed in both males and females.

**Table 5:** Temperature gradients (∇T) from body core to the skin (∇T_r-s_)^1^ and for the skin to the environment (∇T_s-a_) under thermoneutral and heat stress conditions.

Gradient (°C)	Condition^2^		Sex		*P*-value		
	TN	HS	Male	Females	Condition	Sex	I^3^
Core to Face	3.50 ± 0.16e	1.62 ± 0.16e	2.53 ± 0.16e	2.58 ± 0.16e	< 0.0001	0.83	0.42
Core to Eye	6.61 ± 0.23d	4.85 ± 0.23d	5.85 ± 0.23d	5.61 ± 0.23d	< 0.0001	0.48	0.81
Core to Breast	2.01 ± 0.29f	−0.22 ± 0.29f	1.11 ± 0.29f	0.68 ± 0.29f	< 0.0001	0.33	0.52
Face to Air	14.3 ± 0.20b	10.1 ± 0.20b	12.4 ± 0.20b	12.0 ± 0.20b	< 0.0001	0.21	0.85
Eye to Air	11.21 ± 0.26c	6.92 ± 0.26c	9.10 ± 0.26c	9.02 ± 0.26c	< 0.0001	0.82	0.66
Breast to Air	15.8 ± 0.32a	12.0 ± 0.32a	13.8 ± 0.32a	13.9 ± 0.32a	< 0.0001	0.83	0.94
***P-value***^4^	< 0.001	< 0.001	< 0.001	< 0.001			

1 The facial, eye, and breast temperatures are defined in Fig. 2.

2 Thermoneutral (TN): 22°C, heat stress (HS): 30°C and 40 – 45 % relative humidity for 10 h/day from d 28 to d 33.

3 *P*-value of the condition-by-sex interaction effect on temperature gradients.

4 *P*-value of the intra-condition and intra-sex differences between temperature gradients. Different letters (a - f) indicate significant differences between gradients presented in the same column.

## Discussion

In HS research, body temperature is a key parameter measured repeatedly to evaluate bird response to elevated T_a_. The aim of the present work is to investigate the range of variation in T_r_ and T_s_ in broiler chickens kept under TN and HS conditions during the finisher phase. These parameters can then be used to assess broilers’ physiological response and thermoregulatory capacity when exposed to high T_a_. The reasoning here is that without knowledge of the full range of variation in body temperature under TN conditions, meaningful conclusions about bird thermotolerance under HS conditions cannot be clearly made due to the potential inter-individual variability in response to exposure to high T_a_. This work also aims to analyse the relationship between T_r_ and T_s_ and to understand changes in this relationship across these different environmental conditions.

In this study, a moderate cyclical HS program was applied from d 28 to d 33. As can be seen in Fig. 3, the target stress temperature of 30°C and relative humidity of 40 - 45 % were successfully achieved, exposing the birds to moderate HS for 10 h per day from 6:00 AM to 4:00 PM.

The application of the above-mentioned HS program led to a significant decrease in feed intake compared to TN conditions. This decrease in feed intake is an adaptive response in birds exposed to high T_a_ as they attempt to decrease their metabolic heat production to prevent further rises in core body temperature (Andretta et al., 2021; Yehia et al., 2024). Consequently, the diminished feed intake contributes to a decrease in BW and an increase in FCR (Andretta et al., 2021; Emami et al., 2021).

Regarding T_r_, the significant increases observed in birds exposed to HS conditions relative to those kept under TN conditions align with literature findings, which show elevated T_r_ following exposure to T_a_ higher than the upper critical limit of the thermoneutral zone (Alhenaky et al., 2017). Additionally, the higher average male T_r_ values compared to females likely reflect greater male metabolic heat production due to higher BW (Andretta et al., 2021).

The wider range of ΔT_r_ values reported under HS conditions suggest that the response to HS varied between birds. In fact, T_r_ values under TN and HS overlapped (Fig. 5), suggesting that some birds were able to maintain their T_r_ in the TN physiological range whilst under HS (Table 3). This variability is of interest as it implies that some birds were able to continue to effectively regulate their core body temperature despite HS conditions. Future studies should aim to elucidate the mechanisms underlying this variability in the response to HS and to potentially identify markers that could be used to select broiler chickens with inherently higher resistance to elevated temperatures.

As for T_s_, Nääs et al. (2010) reported a narrower range of T_se_ values compared to those from the present study, which ranged from 38.4 ± 0.4 to 39.6 ± 0.5°C (ΔT_se_ = 1.2°C), while T_sf_ varied between 37.8 ± 0.7 to 39.4 ± 0.9°C (ΔT_sf_ = 1.6°C) at d 42 under T_a_ varying between 25.4 and 31.9°C. In a different study, researchers recorded T_s_ between d 8 and d 36 of commercial broiler chickens kept under TN and HS conditions and reported T_sf_ values that ranged from slightly above 36°C to almost 42°C under T_a_ between 22 and 38°C (Giloh et al., 2012). Between-study variability in temperature data obtained using infrared thermography could be plausibly attributed to several factors and their combinations, including: 1) differential inter-subject response to variations in T_a_ (as previously discussed), 2) differences between experimental thermography protocols (e.g., region of interest, angle of camera, distance between camera lens and bird, temperature extraction method, and thermal calibration), and 3) differences in experimental design such as bird strain and HS regimen.

The significant increase in T_s_ under HS indicates a greater heat load under stress conditions (Lin et al., 2005a, 2005b; Nääs et al., 2010). The elevation in T_s_ occurs due to vasodilation, which leads to the redirection of blood flow from the core to the periphery (Giloh et al., 2012). This process facilitates heat dissipation into the environment through radiation, convection, and conduction to ensure stable thermoregulation. Moreover, we observed that the interaction between thermal conditions and the anatomical regions was not statistically significant while the main effect of the anatomical region was highly significant. This indicates that differences in T_s_ between different anatomical regions are consistent across environmental conditions (i.e., all regions contribute to passive heat dissipation). This statement is supported by the correlation between all three parameters of T_s_ found in our study. Differences in tissue vascularization and surrounding microenvironment (e.g., T_a_, RH, ventilation, bird density) could plausibly explain the observed differences in T_s_ (Cangar et al., 2008; Nascimento et al., 2011).

In the current study, we also analysed the relationship between T_r_ and T_s_ to understand how this relationship could change under heat challenge. The strength of the correlation between T_r_ and T_s_ varied depending on the environmental condition. They were low to moderate under TN conditions and were moderate to high under HS conditions. This difference in correlation coefficients likely stems from T_s_ being more susceptible to immediate changes in the surrounding environment than T_r_ since the skin is in direct contact with the external environment. In contrast, T_r_ remains relatively more resistant to change due to homeostatic mechanisms such as basal metabolism and blood circulation, which are less affected by immediate external conditions. When data obtained under both conditions were aggregated together, very strong and positive correlations were found between T_r_ and T_s_. The aggregation of temperature data obtained under both conditions yielded a wider range of values over which the correlations were highly strong and significant. The relationship between T_r_ and T_s_ is of great interest, as it allows us to evaluate the thermoregulatory status of broiler flocks under HS in a non-invasive manner during critical time periods (e.g., summer heat waves), which often have devastating impacts on broiler performance, health, and welfare.

Results of the present study showed that, under TN conditions, the heat transfer rate from the core to the eye (∇T_r-se_) was higher than that to the face (∇T_r-sf_) and to the breast skin (∇T_r-sb_). Despite lower ∇T_r-sb_, our findings demonstrate that more heat was being transferred from the breast skin to the air than from the face or the eye as indicated by the significantly higher ∇T_sb-a_ compared with ∇T_sf-a_ and ∇T_se-a_.

These patterns of heat transfer from the core to the surface and from the surface to the air were also found under HS conditions. They could suggest conserved and differential contributions of these anatomical regions to overall heat dissipation. Further research is needed to confirm these trends and to understand their thermoregulatory importance.

All ∇T_r-s_ and ∇T_s-a_ were significantly lower under HS than under TN, which is in line with findings from previous studies (Lin et al., 2005a, 2005b). The decrease in ∇T under HS conditions indicates a reduced heat transfer from the core to the periphery and then to the air under higher T_a_, leading to increased heat load in the birds (Lin et al., 2005a, 2005b).

According to these findings, heat transfer to the eyes seems to be prioritized under both TN and HS conditions. However, heat dissipation to the environment seems to be determined by the surface area of the anatomical region, with the breast skin playing a more pronounced role than the face and the eyes. An earlier study in laying hens showed that total blood circulation increased from 53 % under TN conditions to 85 % under HS, through the arteriovenous anastomoses of unfeathered skin, thereby bypassing the capillary bed and enhancing heat transfer from internal organs to the periphery and, ultimately, to the environment (Wolfenson, 1983). Additionally, when birds are exposed to persistent HS, they exhibit behavioural changes to further promote heat dissipation. For instance, Cartoni Mancinelli et al. (2023) showed that roosting behaviour (i.e., laying down with the ventral body region in contact with the litter) significantly increased when T_a_ rose from 26°C to 30°C. This increased contact between the ventral skin area and the litter support the important role of the breast skin as it could further facilitate environmental heat transfer by conduction.

While this study provides insights regarding Ross 308 broiler heat transfer dynamics under a specific HS protocol (30°C, 40-45 % RH), limited conclusions can be drawn regarding other broiler strains subjected to diverse HS conditions. Further research using multiple broiler strains and varied stress protocols can increase available data regarding thermoregulation under HS. Moreover, taking daily thermal images before and after stress application will help elucidate broiler acclimatization to HS and provide a more comprehensive account of inter-day and inter-individual differences in heat transfer dynamics. Further research is also required to understand heat transfer dynamics in birds that continue to exhibit rectal temperatures in the thermoneutral physiological range whilst under heat stress conditions.

## Conclusions

In the context of increasing global temperatures due to climate change, heat stress represents a major challenge for poultry production. Here, we confirmed that both rectal and surface temperatures were more variable under HS than under TN. Our study also showed that heat transfer from and to different anatomical regions of the skin significantly differed under both TN and HS conditions. Heat was transferred at a higher rate from the core to the eyes than to the face and the breast skin under TN and HS. However, the breast skin transferred heat to the environment at a higher rate than the face and the eye under both conditions, which is probably due to its greater surface area. Finally, this study reiterated the potential of infrared thermography to be used to monitor core body temperature in a contactless and stress-free manner for the detection of HS indicators in poultry based on the strong correlation between T_r_ and T_s_ reported in this study. Future research should focus on understanding the determinants of the variability in the response of broilers to HS and on elucidating heat transfer dynamics in birds that exhibit body temperature in the TN physiological range despite being under HS conditions.

## Declaration of competing interest

The authors declare the following financial interests/personal relationships which may be considered as potential competing interests:

Nabeel Alnahhas reports financial support was provided by Quebec's Ministry of Agriculture, Fisheries and Food. Nabeel Alnahhas reports financial support was provided by Natural Sciences and Engineering Research Council of Canada. Jean-Michel Allard Prus reports a relationship with Scott Hatchery (Quebec, Canada) that includes: employment. If there are other authors, they declare that they have no known competing financial interests or personal relationships that could have appeared to influence the work reported in this paper.
